# Transcriptome sequencing reveals novel *Citrus bark cracking viroid* (CBCVd) variants from citrus and their molecular characterization

**DOI:** 10.1371/journal.pone.0198022

**Published:** 2018-06-11

**Authors:** Yafei Wang, Sagheer Atta, Xuefeng Wang, Fangyun Yang, Changyong Zhou, Mengji Cao

**Affiliations:** 1 National Citrus Engineering Research Center, Citrus Research Institute, Southwest University, Chongqing, China; 2 Academy of Agricultural Sciences, Southwest University, Chongqing, China; 3 Department of Plant Protection, Faculty of Agricultural Sciences, Ghazi University, Dera Ghazi Khan, Pakistan; Birla Institute of Technology and Science, INDIA

## Abstract

*Citrus bark cracking viroid* (CBCVd), previously called *Citrus viroid IV*, belongs to the genus *Cocadviroid* within the family *Pospiviroidae*. CBCVd has been identified as an important causative agent in citrus and hops. In this study, we obtained the full-length genomes of different variants of all detected citrus viroids from Pakistan through transcriptome sequencing. Different CBCVd variants were first found in Pakistan. These newly discovered Pakistani CBCVd variants were provisionally called “CBCVd-LSS” for their low sequence similarity (80.9%–88.9%) with the CBCVd RefSeq sequence (NC_003539). The two most predominant CBCVd sequences from Pakistan had the closest identity, 90.6% and 87.9%, with two CBCVd sequences isolated from hops. Identification and molecular characterization of CBCVd from citrus in Pakistan and China were also reported. The length of CBCVd from China ranged from 282 to 286 nucleotides, while that of the one from Pakistan ranged from 273 to 277 nucleotides. Based on genetic diversity and phylogenetic analysis, two main CBCVd clades were identified. CBCVd sequences from Pakistan, China, and other countries were further divided into six sub-clades. Sequence alignment revealed some nucleotide changes between these sub-clades, and analysis indicated that several mutations could significantly affect the primary and secondary structure of the viroid. Our results indicated that the CBCVd sequences from Pakistan and China were significantly different with respect to genome and secondary structure and Pakistan might be one of the independent geographical origins of CBCVd worldwide.

## Introduction

Viroids, the known smallest pathogens, are closed-loop, single-stranded RNA molecules of 246–401 nucleotides (nt) [1, 2]. Eight viroid species of the family *Pospiviroidae*: *Citrus exocortis viroid* (CEVd), *Citrus bent leaf viroid* (CBLVd), *Hop stunt viroid* (HSVd), *Citrus dwarfing viroid* (CDVd), *Citrus bark cracking viroid* (CBCVd), *Citrus viroid V* (CVd-V), *Citrus viroid VI* (CVd-VI) and *Citrus viroid VII* (CVd-VII) have been found on citrus trees [3–6].

CBCVd was formerly known as *Citrus viroid IV* (CVd-IV). It is located in the nucleus and its rod-shaped secondary structure has a central conserved region (CCR) and a terminal conserved hairpin (TCH). CVd-IV has been associated with severe bark cracking of trifoliate and trifoliate citrus hybrids and it was renamed as CBCVd according to its symptoms on trifoliate orange [7]. CBCVd has been identified as a critical component of citrus viroid mixtures that can induce severe exocortis-like bark scaling symptoms on trifoliate orange rootstock with no CEVd [8, 9]. CBCVd has a broad host range among citrus, citrus relatives, and citrus hybrids as well as herbaceous hosts such as cucumber, tomato, eggplant, datura, chrysanthemum, and gynura [10]. Recently, it has been also reported that plant growth and crop yield will be significantly affected when hops (*Humulus lupulus* L.) are infected with CBCVd [11, 12]. CBCVd can cause severe stunting disease in hops, which is one of the most devastating diseases in this plant [11, 12].

The Pakistani region is an important citrus cultivation area in the world. However, due to the special geographical location, the study of citrus viroids in the region is not sufficient. The occurrence of the CBCVd has never been reported in Pakistan where CEVd, CBLVd, HSVd, CDVd and CVd-V have been detected in citrus [13, 14]. Fortunately, our lab had a rich collection of citrus samples from Pakistan in the past decade. An overall analysis of citrus viroids in Pakistan will help us understand the occurrence of viroid conditions in the Pakistani region and may lead to the discovery of new viroids. In China, many citrus species have been imported from other courtries such as Japan. However, information on the existence of CBCVd in these introductions is still pending.

In recent years, next-generation sequencing (NGS) platforms combined with bioinformatics analysis methods have been widely used in viroid research and diagnosis for viroid discovery, detection, and viroid-host interactions [15–18]. The utilization of NGS in viroid research has led to the discovery of new viroids, such as *Grapevine latent viroid* and *Persimmon viroid 2* [19, 20]. Hops infected with CBCVd variants and fig trees infected with *Apple dimple fruit viroid* were also discovered with the help of NGS [11, 21]. NGS has also provided an alternative method of identifying viroids in citrus cultivars. Here, the identification of different viroids using transcriptome sequencing from citrus plants is described and the novel CBCVd variants found in citrus cultivars were further assessed using RT-PCR analysis. We further described molecular characterization of CBCVd variants from citrus in Pakistan and China. Sequence diversity and the phylogenetic relationship of the CBCVd isolates from different regions are also reported here. The secondary structures of different CBCVd sequences were also here analyzed in this study.

## Materials and methods

### Ethics statement

All experimental citrus materials were collected from the greenhouse of National Citrus Engineering Research Center, Citrus Research Institute, Southwest University, in Beibei District, Chongqing municipality, China. There was no specific permission required for these collection activities because only a preliminary investigation of the disease status of the preserved citrus materials was undertaken. We confirmed that the collection did not involve endangered or protected species.

### Plant materials

All citrus samples were collected from May 2008 to April 2017 in main citrus-growing regions of Punjab Province, Pakistan and Zhejiang Province, China. In total, 93 and 184 citrus samples from Pakistan and China were collected, respectively. Budwoods from these samples were grafted onto rough lemon rootstocks and nurtured in the pest-control greenhouse of National Citrus Engineering Research Center, Chongqing, China.

### Transcriptome sequencing and bioinformatics analysis

Total RNA was extracted using the EASY spin Plus Complex Plant RNA Kit (Aidlab Biotech, Beijing, China). The transcriptome libraries were constructed from two rRNA-depleted libraries using a TruSeq RNA-seq Sample Preparation Kit (Illumina, San Diego, CA, US). The libraries were sequenced using an Illumina HiSeq X-ten platform with a paired-end 150 bp set-up. Two sets of sequences, PCV-I and PCV-II, were finally obtained. PCV-I library had 43 Pakistani samples and PCV-II library had the other 40 Pakistani samples. We collected approximately 25G transcriptomic data for analysis from PCV-I and PCV-II, respectively. All raw sequences were used for mapping to the reference genomes of sweet orange (*C*. *sinensis*) [22] and clementine (*C*. *clementina*) [23] to remove the reads originating from hosts using CLC Genomics Workbench 9.5 (Qiagen, Denmark). The remaining reads were *de novo* assembled into contigs (transcripts) with the minimum of 200 nt and then used for BLASTN searches against an all-viroid-sequence database.

### RT-PCR, cloning, and sequencing

All the samples from Pakistan and China were analyzed in the usual fashion, by RT-PCR. Primer pairs of CBCVd (CBCVd-F: 5′-GGGGAAATCTCTTCAGAC-3′, CBCVd-R: 5′-GGGGATCCCTCTTCAGGT-3′) [24], which were previously reported, were used to amplify CBCVd from these samples. RT-PCR products were first confirmed using direct sequencing and then cloned into pGEM-T Easy Vector (Promega, China) according to the manufacturer’s instructions. The insert was transferred into *Escherichia coli* DH5α cells. Recombinant DNA clones containing inserts of the expected size were identified by PCR. At least 15 clones were randomly chosen from every CBCVd isolate and sent to TsingKe (Chengdu, China) for sequencing.

### Genetic diversity, phylogenetic analysis, and determination of secondary structures

The program Clustal W was used to perform the alignment of multiple sequences. Genetic distance analysis about CBCVd isolates was performed using the MEGA 6 software with the Jukes-Cantor Model. Phylogenetic analysis was performed using maximum likelihood phylogeny with 1000 bootstrap replications. Values below 70% were cut off. The MFOLD web server and RNAviz program were used to obtain stable secondary structure. Sequences from this study and other sequences obtained from GenBank were used in the analysis (S1 Table).

## Results

### Pathogen identification

The Illumina sequencing generated 244,781,032 and 223,983,226 raw paired-end 150 bp reads for PCV-I and PCV-II libraries, respectively. After mapping to two high-quality reference genomes of hosts, 39,861,968 and 37,884,631 unmapped reads were obtained from PCV-I and PCV-II libraries, respectively. These unmapped reads were *de novo* assembled into contigs with a minimum length of 200 nt. Finally, 29,499 and 30,501 contigs were generated and used for BLASTN searches. BLASTN searches revealed the presence of contigs of six different citrus viroids except CVd-VI and CVd-VII in both samples. The full genomes of six viroids including CEVd, CBLVd, HSVd, CDVd, CBCVd, and CVd-V were obtained from PCV-I and PCV-II libraries. Different contigs of these citrus viroids were also found in PCV-I and PCV-II libraries by transcriptome sequencing and BLASTN searches. Finally, we found one CEVd contig, one CBLVd contig, one CVd-I-LSS contig, one HSVd contig, two CDVd contigs, one CBCVd contig, and two CVd-V contigs in the PCV-I library. And we obtained one CEVd contig, one CBLVd contig, one HSVd contig, two CDVd contigs, one CBCVd contig and one CVd-V contig from the PCV-II library. Two CBCVd contigs were found in the PCV-I and PCV-II libraries, with an assembled length of 277 and 276 nt, respectively. Average coverage of reads mapping for these contigs were 19.43–394.38 and 20.84–627.66 in the PCV-I and PCV-II libraries, respectively (Table 1).

**Table 1:** Blast results of contigs for samples (PCV-I, PCV-II, respectively) against viroid sequences available in NCBI.

Sample	Accession number	Reference name	Reference length	Contig name	Contig length	Identity (%)	Total read count	Average coverage
PCV-I	KC290927.1	CEVd	371	Contig-412	371	99	511	204.85
	FJ773267.1	CBLVd	328	Contig-5366	327	99	43	19.43
	KF726094.1	CVd-I-LSS	328	Contig-5367	328	98	151	69.01
	AY594203.1	HSVd	299	Contig-1830	299	99	795	394.38
	FJ773277.1	CDVd	293	Contig-8134	293	99	94	47.96
	AB054619.1	CDVd	294	Contig-8135	294	100	249	126.95
	KM211546.1	CBCVd	284	Contig-6751	277	91	118	62.94
	JQ348927.1	CVd-V	293	Contig-818	293	100	476	240.59
	AB560862.1	CVd-V	294	Contig-820	294	99	219	111.15
PCV-II	FJ904297.1	CEVd	371	Contig-4665	371	100	126	50.23
	KM214213.1	CBLVd	327	Contig-7695	327	100	558	254.68
	HE662806.1	HSVd	299	Contig-4624	300	97	244	120.54
	AJ630358.1	CDVd	294	Contig-2719	294	99	196	98.98
	AF447788.1	CDVd	292	Contig-2720	292	99	112	57.44
	KM211547.1	CBCVd	284	Contig-12528	276	88	39	20.84
	EF617306.1	CVd-V	294	Contig-902	294	99	1237	627.66

### Confirmation and analysis of CBCVd from Pakistan

**Fig 1:**
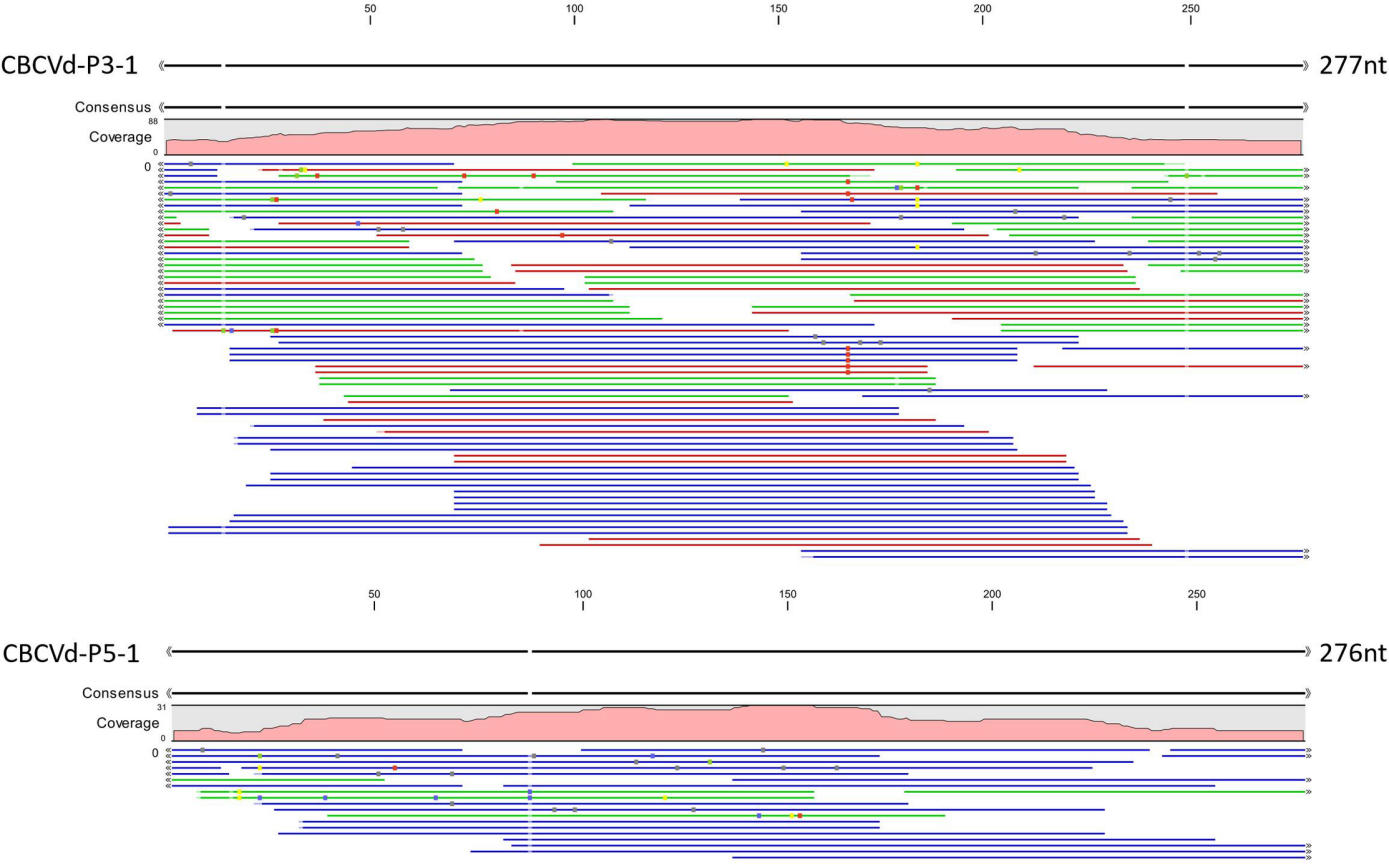
**Transcriptome reads mapped onto sequences of CBCVd-P3-1 (A) and P5-1 (B) in PCV-I and PCV-II libraries.** Dots represent mismatches of reads.

An RT-PCR screening assay for CBCVd from Pakistan was also performed and the results showed that 6 out of 93 samples were CBCVd-positive. Cloning and sequencing of the RT-PCR products showed the presence of Pakistani CBCVd variants, and their genomes were subsequently confirmed. A sequence was considered to be the predominant sequence if it appeared twice or more in the same host. Based on these data, it was speculated that at least eight Pakistani CBCVd sequences were predominant. One predominant CBCVd sequence (P3-1, Accession number: MG457780) found in PCV-I and another predominant CBCVd sequence (P5-1, Accession number: MG457778) found in PCV-II were the two most predominant sequences. Our results also showed the lengths of P3-1 and P5-1 to be 277 nt and 276 nt, respectively. P3-1 and P5-1 had only 88.5% and 86.2% nucleotide sequence identity with the CBCVd RefSeq sequence (NC_003539), respectively, and 95.0% nucleotide sequence identity with each other. Total read count and average coverage of reads mapped onto sequences of CBCVd-P3-1 and P5-1 were 118, 62.94 and 39, 20.84 in the PCV-I and PCV-II libraries, respectively (Fig 1).

### Genetic diversity of CBCVd isolates from Pakistan and China

In order to confirm whether there were similar CBCVd isolates in China, we further analyzed citrus samples from Pakistan and China using RT-PCR. Finally, six CBCVd isolates (P1–P6) from Pakistan and eight CBCVd isolates (C1–C8) from China were found. These CBCVd isolates were amplified by RT-PCR and at least 15 cDNA clones were randomly selected from each of the 14 CBCVd isolates for sequencing. We performed PCR amplification using high-fidelity polymerase to minimize errors and confirmed the mutations in different clonal sequences to ensure they were consistent. Sequencing analysis showed the length of CBCVd sequences in Pakistan and China to be 272–277 nt and 282–286 nt, respectively (Table 2). These Pakistani CBCVd sequences had only 80.9% to 88.9% nucleotide sequence similarities to the CBCVd RefSeq sequence (NC_003539). The nucleotide sequence similarities among the Pakistani and Chinese isolates were 88.5–100% and 90.6–100%, respectively.

**Table 2:** Genomic diversity of *citrus bark cracking viroid* (CBCVd) isolates from Pakistan and China.

Name	Original host	No. of clones sequenced	Predominant sequence	Singleton sequence	C	V	Pi	S	N	Length (nt)
P1	Mosambi	19	2	11	253	23	1	22	0.011±0.003	273, 276
P2	Saccari	19	2	11	260	17	3	14	0.008±0.002	275, 276, 277
P3	Feutrell’s Early	18	1	10	267	10	1	9	0.005±0.002	275, 276, 277
P4	Saccari	20	1	16	248	29	2	27	0.012±0.002	275, 276, 277
P5	Mosambi	20	1	13	257	19	1	18	0.008±0.002	273, 275, 276, 277
P6	Mosambi	16	1	13	258	19	6	13	0.014±0.004	275, 276, 277
C1	Oostu No.4	23	2	18	262	24	9	15	0.014±0.004	284, 285, 286
C2	Shiranuhi	16	1	12	268	18	4	14	0.011±0.003	283, 284, 286
C3	Katsuyama Iyokan	18	1	10	271	13	1	12	0.006±0.001	282, 284, 285
C4	Yura	18	3	10	268	18	5	13	0.011±0.003	282, 284, 285, 286
C5	Akemi	16	2	9	264	22	10	12	0.019±0.004	284, 286
C6	Nishirkaori	26	2	21	264	22	10	12	0.013±0.003	283, 284, 285, 286
C7	Akemi	16	2	10	258	26	1	25	0.013±0.003	282, 283, 284, 285
C8	Nishirkaori	19	1	15	266	20	5	15	0.010±0.003	284, 286

C, conserved sites; V, variable sites; Pi, parsimonious informative nucleotide sites; S, singleton sites; N, nucleotide diversity

The genetic diversity was further analyzed using 264 CBCVd sequences obtained in this study and 26 other CBCVd sequences including two sequences from the hop available in the NCBI. All of these sequences were small, hand-trimmed, and aligned by the Clustal W program. Finally, 292 nucleotide sites were found. Of the 155 variable sites obtained, there were 74 and 81 parsimoniously informative singletons. Two or more clones had the same mutation in parsimoniously informative sites. Twenty-two predominant CBCVd sequences from Pakistan and China were obtained. The genetic diversity of the Pakistani CBCVd population was 0.027±0.006, which was higher than the Chinese population (0.026±0.005) and other countries’ population (0.022±0.005). When all the CBCVd sequences were considered regardless of geographic regions, the nucleotide diversity was 0.052±0.010 (Table 3).

**Table 3:** Nucleotide diversity of *citrus bark cracking viroid* (CBCVd) sequences from Pakistan, China, and other countries^a^.

Origin	Original host	No. of isolates examined	No. of clones sequenced	C^b^	V^c^	Pi^d^	S^e^	N^f^
Pakistan	Citrus	6	112	190	89	28	61	0.027±0.006
China	Citrus	8	150	189	98	36	62	0.026±0.005
Others	Citrus, Hop	26	26	260	26	17	9	0.022±0.005
All	Citrus, Hop	40	288	137	155	74	81	0.052±0.010

^a^ other countries including Japan, Cuba, Iran, South Africa, Greece, Slovenia, Cyprus and Israel;

^b^ conserved sites;

^c^ variable sites;

^d^ parsimonious informative nucleotide sites;

^e^ singleton sites;

^f^ nucleotide diversity

### Phylogenetic analysis of CBCVd

**Fig 2:**
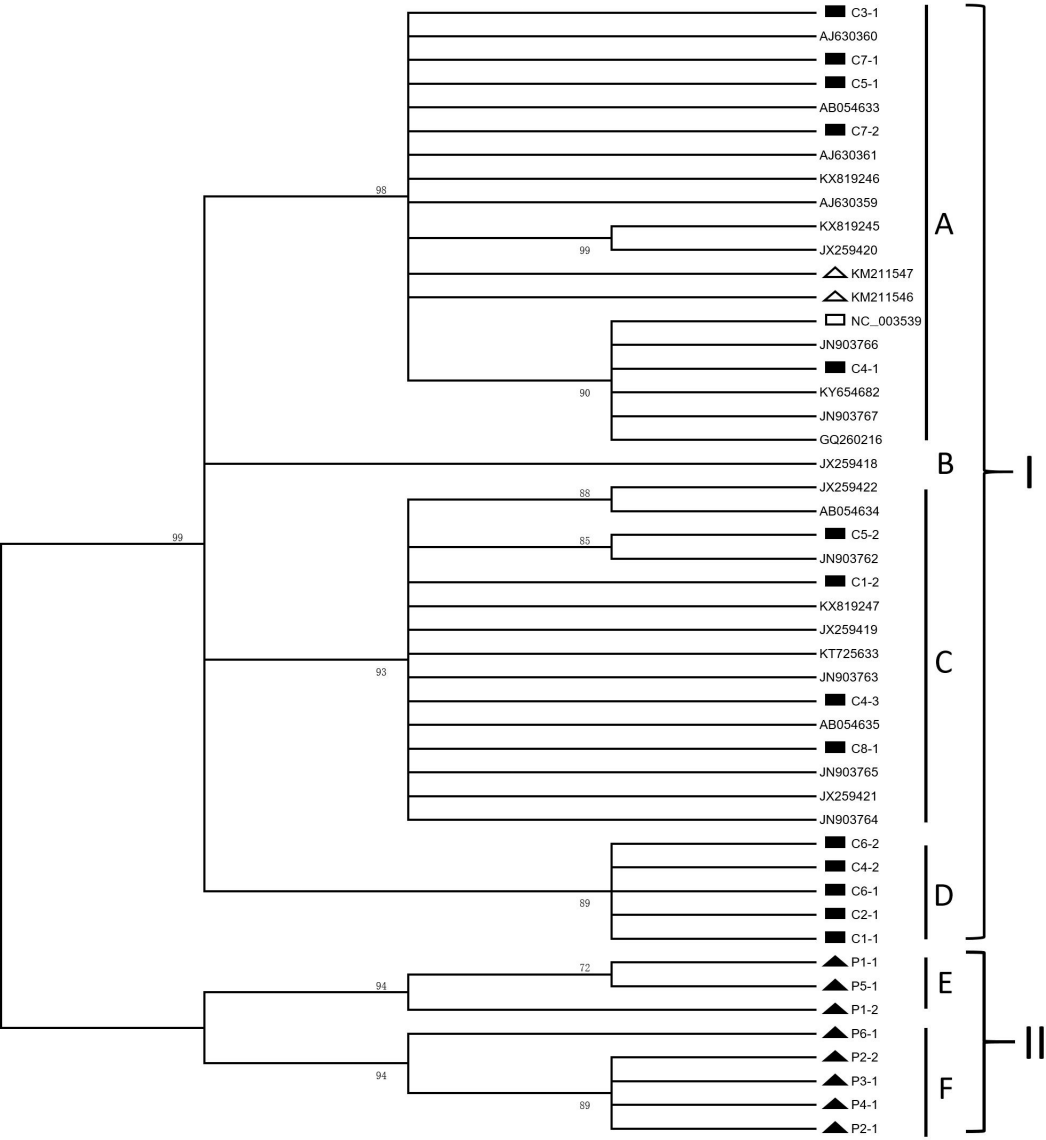
Phylogenetic analysis of *citrus bark cracking viroid* (CBCVd) sequences. The phylogenetic tree was constructed with genomic sequences of 8 Pakistani CBCVd sequences (indicated by solid triangle), 14 Chinese CBCVd sequences (indicated by solid rectangle), and 26 CBCVd sequences including two variant sequences on hop (indicated by hollow triangle) and the CBCVd RefSeq sequence (indicated by hollow rectangle) available in GenBank. Values below 70% were cut off.

The phylogenetic relationships of the CBCVd sequences were analyzed using the 8 predominant CBCVd sequences from Pakistan and 14 predominant CBCVd sequences from China obtained in this study and 26 other CBCVd sequences available in the NCBI. The 48 CBCVd sequences in the phylogenetic tree were separated into two major clades according to geographical regions. Chinese CBCVd sequences were placed in the largest clade while Pakistani CBCVd sequences were placed in the other clade. Phylogenetic analysis showed that Pakistani CBCVd sequences formed an individual clade distinct from the other clade. No intermediate CBCVd sequences were able to connect Pakistani CBCVd sequences and the CBCVd sequences from other countries, including China. The CBCVd sequences were further separated into sub-clades A, B, C, D, E, and F with sequence homology of less than 97.0% between each two sub-clades (Fig 2).

### Secondary structure analysis of putative CBCVd groups

**Fig 3:**
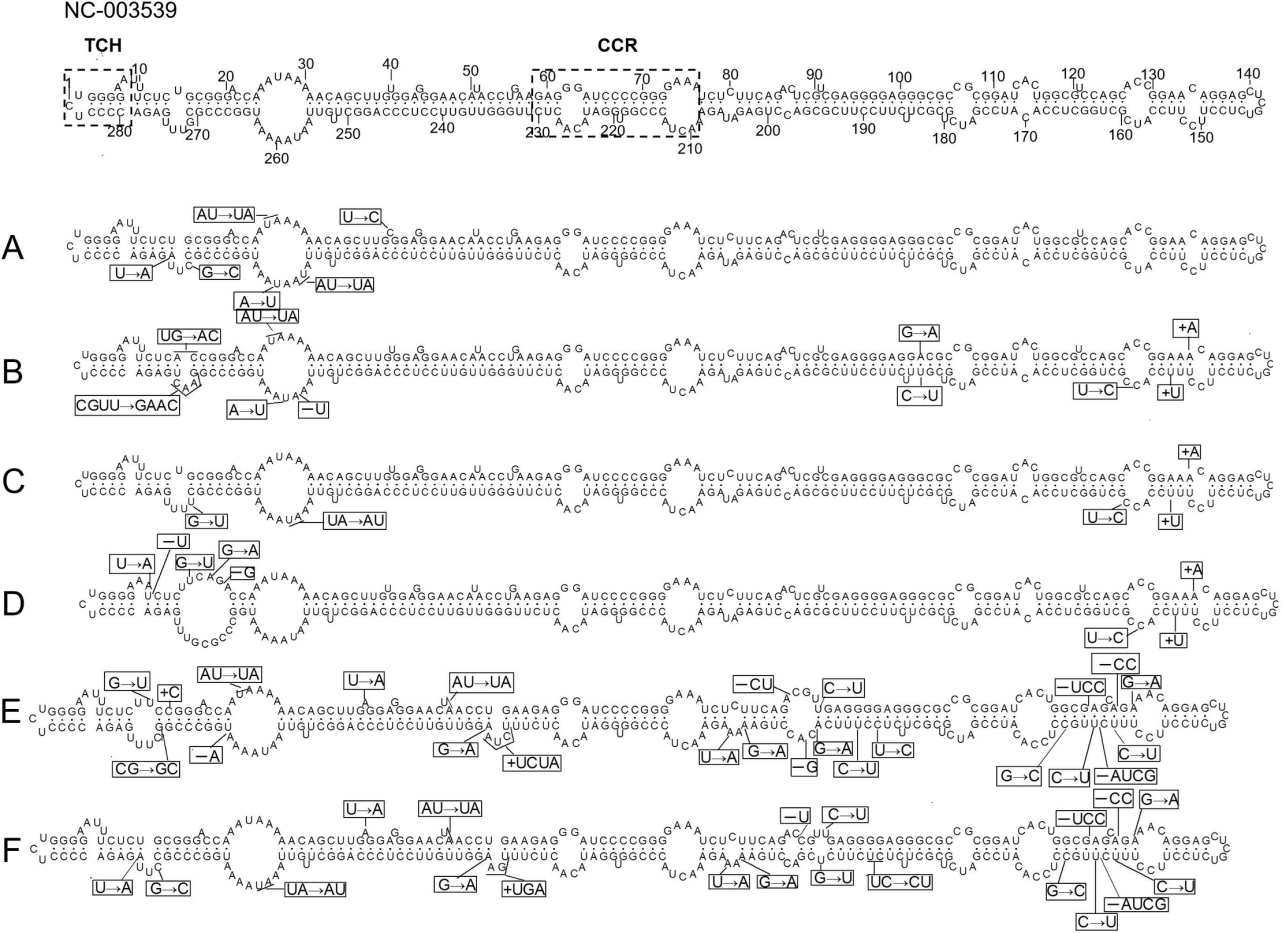
Primary and secondary structures of *citrus bark cracking viroid* (CBCVd) sequences from sub-clades A, B, C, D, E, and F and CBCVd reference sequence (NC003539). The mutations of CBCVd sequences from these sub-clades were shown in box.

The most predominant sequences were selected from every sub-clade for secondary structure analysis of putative CBCVd groups. The terminal conserved hairpin (TCH) (^280^CCCCUCUGGGGAA^8^) and central conserved region (CCR) (^59^GAGGGAUCCCCGGGGAAA^76^ and ^210^ACUACCCGGUGGAUACAACUC^230^) were conserved in all CBCVd sequences. In the secondary structure, the mutations (G^16^→U, G^18^→A, and^20^-U) in sub-clade D extended one loop and the deletion of ^10^U reduced the other loop. Compared to the CBCVd RefSeq sequence (NC_003539), three-stage stable base deletion mutations (^121^-UCC^123^, ^128^-CC^129^, ^157^-AUCG^160^) of the Pakistani sequences in sub-clades E and F were found. They led to the shortening of the genome and the significant change of some domains. The insertion mutations, ^232^+UCGA^233^ in sub-clade E and ^232^+UGA^233^ in sub-clade F, directly created two unique bulge structures. The substitution mutation (AU^50-51^→UA) that occurred in sub-clades E and F also changed one little bulge. Ten stable mutations (U^40^→A, AU^50-51^→UA, C^92^→U, G^131^→A, C^155^→U, C^161^→U, G^164^→C, G^203^→A, U^205^→A, G^233^→A) were confirmed in sub-clades E and F. Sixteen other mutations (G^16^→U, AU^26-27^→UA, ^17^+C^18^, ^87^-UU^88^, ^88^-U, U^188^→C, C^190^→U, G^194^→A, G^194^→U, UC^188-189^→CU, ^196^-G, ^263^-A, UA^258-259^→AU, CG^271-272^→GC, G^272^→C, U^275^→A) were also found in different Pakistani sequences. They also somewhat affected the genome and secondary structure of the Pakistani CBCVd (Fig 3).

## Discussion

Recently, various NGS technologies have come to be widely used in different viroid studies. A variety of sequencing platforms have revolutionized the detection and discovery of plant viroids [18, 25]. Notably, sRNA deep sequencing has been widely adopted to detect plant viroid [11, 21]. Previous studies have shown that the rRNA-depleted total RNA method is generally more effective than the sRNA method when reassembling sequencing reads [25]. However, there are few reports on the identification of new viroids by transcriptome sequencing. In this study, deep sequencing of rRNA-depleted total RNA firstly helped us in obtaining the full-length genomes of all detected known citrus viroids including CEVd, CBLVd, HSVd, CDVd, CBCVd, and CVd-V from Pakistani samples. The different contigs of these citrus viroids were also identified and we found two or more contigs for each detected viroid using deep sequencing of rRNA-depleted total RNA and BLASTN searches. These results demonstrated the advantage of transcriptome sequencing that allows simultaneous identification of some different viroids from various samples. This identification method, focusing on transcriptome sequencing, has been proved to be a useful tool in citrus viroid diagnostics.

This study reported the identification of CBCVd in Pakistan, where this pathogen was found for the first time. We also got the Pakistani CBCVd sequences, which differed from the reported CBCVd sequences. Two most predominant CBCVd sequences (P3-1 and P5-1) from Pakistan had the highest identity (90.6% and 87.9%) with two CBCVd sequences (Accession number in NCBI: KM211546 and KM211547) isolated from hops. These novel Pakistani CBCVd sequences were temporarily named “CBCVd-LSS” (tentative acronym) because they have low sequence similarity (80.9%–88.9%) to the CBCVd RefSeq sequence (NC_003539). The level of 90% sequence similarity was usually the definitive boundary that differentiates strains and species [26, 27]. In case of the CBCVd-LSS and CBCVd sequences, it was difficult to distinguish them from each other with only nucleotide sequence homology. We did not find intermediate sequences that connected the populations of Pakistani CBCVd sequences to those of CBCVd sequences from other countries in this study. Because of the discontinuity in genomic variation between CBCVd-LSS and CBCVd, we thought CBCVd-LSS might be a new species, distinct from CBCVd. More likely, CBCVd-LSS was only a unique strain of CBCVd. In this way, more work is needed to accurately classify CBCVd-LSS and CBCVd. Biological properties of CBCVd-LSS will need to be determined in the following study. If there is no difference in the biological characteristics (host range, symptoms) between CBCVd-LSS and CBCVd, CBCVd-LSS can be considered a unique strain of CBCVd.

In the past, no CBCVd was detected in Pakistan, probably because of the low occurrence frequency and high variation frequency of the Pakistani CBCVd isolates. The fact that there were several sequence variations between CBCVd-LSS and CBCVd sequences in the vicinity of the primers explains why no specific products were obtained by RT-PCR in previous studies [13, 14, 28]. Similarly, the presence of the same nucleotide sequence in the primer region explains why the primer pair used in this study could be amplified by RT-PCR to produce specific fragments of CBCVd-LSS and CBCVd. CBCVd was identified with a low incidence in Pakistan (6 of 93) and in China (8 of 184) in this study. CBCVd has also been detected in Israel, Japan, Greece, and other countries [8, 29, 30]. According to existing reports, citrus varieties and hops are important hosts of CBCVd. Severe crack symptoms were observed in sensitive rootstocks when the rootstocks were infected by mixed viroids even with no CEVd [8, 9]. Infection with CBCVd, especially coinfections with other viroids, could result in substantial losses of citrus production. In addition, CBCVd could seriously affect the growth of hops and cause major losses. For this reason, removal of CBCVd should be considered in the breeding of citrus and hops.

So far, the molecular characteristics and evolutionary analysis of several citrus viroids have been reported [14, 28, 31–33]. In this study, CBCVd-LSS was detected in six citrus cultivars from Pakistan. Eight citrus cultivars with CBCVd from China were also detected. The high-fidelity DNA polymerase was used in PCR amplification to ensure that the mutations detected in this study were all from natural mutations. Genetic analysis of CBCVd variants showed the genetic diversity of CBCVd sequences from Pakistan to be more pronounced than that from other countries. Phylogenetic analysis showed that the CBCVd sequences from Pakistan and China were located in different clades. This is the first report that CBCVd sequences from Pakistan were grouped according to the geographic origin. In addition, the CBCVd variants from other Asian countries such as Japan, Iran and Israel were all placed in the clade I, indicating that there were few differences among these CBCVd variants. And the genetic distance between these CBCVd variants and Chinese CBCVd variants was also very close. Although these Asian countries, especially Iran, were close to Pakistan, there were significant differences between the CBCVd sequences from these regions and the Pakistani CBCVd sequences. Sequence analysis showed that few mutations were presented on the CBCVd variants in sub-clades A, B, C, and D, indicating a close relationship among these sub-clades. The eight Chinese cultivars infected with CBCVd were originally imported from other countries such as Japan. These CBCVd variants in sub-clades A, B, C, and D might have the same ancestors. Our results also showed Pakistani CBCVd sequences to differ significantly from the CBCVd sequences from China and other countries. The results of these genetic diversity and phylogenetic analysis indicated that Pakistan might be one of the independent geographical origins of CBCVd worldwide.

By analyzing the putative secondary structures of different CBCVd sequences, sequence variations found in this study were found to affect the viroid structures. Our analysis also suggested that some nucleotide changes were responsible for the structural differences between the CBCVd-LSS sequences and the reference CBCVd sequence. The sequences of CBCVd-LSS were also different and the variations between these sequences also changed CBCVd-LSS structures. Some nucleotide changes in certain regions were also found to cause some other nucleotides changed at complementary positions. The accumulation of these unusual changes eventually separated the CBCVd-LSS and CBCVd populations from each other. Previous studies have suggested that the major functional motifs of viroids were the loops and bulges in their rod-like secondary structures [34]. Some of the changes identified in this study significantly affected the morphology of the secondary structure of the viroids, which suggests that these changes are likely to affect their virulence. The biological effect of these changes will be determined by further infectivity assays.

In conclusion, our study identified and provided an initial characterization of novel Pakistani CBCVd variants here called “CBCVd-LSS” on citrus cultivars by transcriptome sequencing. They are named for their low sequence similarity to the CBCVd RefSeq sequence (NC_003539). We also reported the molecular characterization of CBCVd from citrus in China and found that the CBCVd sequences from China and Pakistan differed significantly in genome and secondary structure. Based on genetic diversity and phylogenetic analysis, we deduced that Pakistan might be one of the independent geographical origins of CBCVd worldwide.

## Supporting information

S1 Table The basic information of *citrus bark cracking viroid* (CBCVd) sequences obtained from this study and GenBank. (DOC)
