# Supplementary material for: Transcriptome sequencing reveals novel Citrus bark cracking viroid (CBCVd) variants from citrus and their molecular characterization
Source: PLoS One. 2018 Jun 11;13(6):e0198022. doi: 10.1371/journal.pone.0198022 (PMC5995356; doi:10.1371/journal.pone.0198022)
Supplement: S1 Table — (DOC) [file pone.0198022.s001.doc]

**S1 Table.** The basic information of *Citrus bark cracking viroid* (CBCVd) sequences obtained from this study and GenBank.

|  | **Variants** | **Original host** | **Geographic origin** | **Accession numbers** | **Reference** |
| --- | --- | --- | --- | --- | --- |
| 1 | P6-1 | Citrus | Pakistan | MG457777 | This work |
| 2 | P5-1 | Citrus | Pakistan | MG457778 | This work |
| 3 | P4-1 | Citrus | Pakistan | MG457779 | This work |
| 4 | P3-1 | Citrus | Pakistan | MG457780 | This work |
| 5 | P2-2 | Citrus | Pakistan | MG457781 | This work |
| 6 | P2-1 | Citrus | Pakistan | MG457782 | This work |
| 7 | P1-2 | Citrus | Pakistan | MG457783 | This work |
| 8 | P1-1 | Citrus | Pakistan | MG457784 | This work |
| 9 | C8-1 | Citrus | China | MG457785 | This work |
| 10 | C7-2 | Citrus | China | MG457786 | This work |
| 11 | C7-1 | Citrus | China | MG457787 | This work |
| 12 | C6-2 | Citrus | China | MG457788 | This work |
| 13 | C6-1 | Citrus | China | MG457789 | This work |
| 14 | C5-2 | Citrus | China | MG457790 | This work |
| 15 | C5-1 | Citrus | China | MG457791 | This work |
| 16 | C4-3 | Citrus | China | MG457792 | This work |
| 17 | C4-2 | Citrus | China | MG457793 | This work |
| 18 | C4-1 | Citrus | China | MG457794 | This work |
| 19 | C3-1 | Citrus | China | MG457795 | This work |
| 20 | C2-1 | Citrus | China | MG457796 | This work |
| 21 | C1-2 | Citrus | China | MG457797 | This work |
| 22 | C1-1 | Citrus | China | MG457798 | This work |
| 23 | LE | Citrus | Japan | AB054633 | (Ito et al. 2002) |
| 24 | MA | Citrus | Japan | AB054634 | (Ito et al. 2002) |
| 25 | MC | Citrus | Japan | AB054635 | (Ito et al. 2002) |
| 26 | Cu86 | Citrus | Cuba | AJ630359 | (Velazquez et al. 2004) |
| 27 | Cu39 | Citrus | Cuba | AJ630360 | (Velazquez et al. 2004) |
| 28 | Cu25 | Citrus | Cuba | AJ630361 | (Velazquez et al. 2004) |
| 29 | IVI1I2 | Citrus sinensis cv. Sanguinelli | Iran | GQ260216 | - |
| 30 | Fortuna | Citrus | South Africa | JN903762 | - |
| 31 | Du Roi | Citrus | South Africa | JN903763 | - |
| 32 | Tarocco | Citrus | South Africa | JN903764 | - |
| 33 | SB Navel | Citrus | South Africa | JN903765 | - |
| 34 | Westin | Citrus | South Africa | JN903766 | - |
| 35 | Gillimberg | Citrus | South Africa | JN903767 | - |
| 36 | 14-1.13-CBCVd | Citrus sinensis (Late Navel Argos navel orange) | Greece: Poros Arboricultural Station | JX259418 | (Wang et al.2013) |
| 37 | 17-1.15-CBCVd | Citrus limon (Novello Athos lemon) | Greece: Poros Arboricultural Station | JX259419 | (Wang et al.2013) |
| 38 | 22-1.20-CBCVd | Citrus limon (Zampetaki lemon) | Greece: Poros Arboricultural Station | JX259420 | (Wang et al.2013) |
| 39 | 23-1.21-CBCVd | Citrus sinensis (Navel Nuclelar PO 25 navel orange) | Greece: Poros Arboricultural Station | JX259421 | (Wang et al.2013) |
| 40 | 22-1.30-CBCVd | Citrus limon (Zampetaki lemon) | Greece: Poros Arboricultural Station | JX259422 | (Wang et al.2013) |
| 41 | HI-1 | Humulus lupulus cv. Celeia | Slovenia | KM211546 | (Jakse et al. 2015) |
| 42 | HI-2 | Humulus lupulus cv. Celeia | Slovenia | KM211547 | (Jakse et al. 2015) |
| 43 | NRCV05 | Citrus sinensis (sweet orange) | Iran | KT725633 | - |
| 44 | CBCVd-CY19 | Citrus sp. | Cyprus | KX819245 | - |
| 45 | CBCVd-CY291 | Citrus sp. | Cyprus | KX819246 | - |
| 46 | CBCVd-CY305 | Citrus sp. | Cyprus | KX819247 | - |
| 47 | PTZ 57R/T | Blood orange cv. Moro | Iran | KY654682 | - |
| 48 | CVd IV | Grapefruit | Israel | NC_003539 | (Puchta et al. 1991) |
